# Effect of dietary glycemic index on insulin resistance in adults without diabetes mellitus: a systematic review and meta-analysis

**DOI:** 10.3389/fnut.2025.1458353

**Published:** 2025-02-13

**Authors:** Yu-Ting Yu, Yu-Hsiang Fu, Yi-Hsien Chen, Yu-Wei Fang, Ming-Hsien Tsai

**Affiliations:** ^1^Department of Medical Education, Shin-Kong Wu Ho-Su Memorial Hospital, Taipei, Taiwan; ^2^Division of Family Medicine, Department of Community Medicine, Landseed International Hospital, Taoyuan, Taiwan; ^3^Division of Geriatrics and Gerontology, Department of Internal Medicine, National Taiwan University, Taipei, Taiwan; ^4^Department of Family Medicine, Shin-Kong Wu Ho-Su Memorial Hospital, Taipei, Taiwan; ^5^Division of Nephrology, Department of Internal Medicine, Hsin-Jen Hospital, New Taipei City, Taiwan; ^6^Department of Medicine, Fu-Jen Catholic University School of Medicine, Taipei, Taiwan

**Keywords:** blood glucose, glycemic index, randomized controlled trial, meta-analysis, homeostasis model assessment of insulin resistance

## Abstract

Low glycemic index (LoGI) diets are associated with decreased insulin resistance and are an effective strategy for patients with diabetes mellitus to control postprandial glucose levels. However, whether these effects on insulin resistance and glucose levels are also observable in those without diabetes mellitus is poorly understood. The present study examined the influence of LoGI diets compared with that of high glycemic index (HiGI) diets on insulin resistance in adults without diabetes mellitus. This meta-analysis included six randomized controlled trials involving 192 participants with a mean age of 52.5 years. A homeostasis model assessment of insulin resistance (HOMA-IR) score was calculated for each trial, and differences in HOMA-IR before and after each dietary intervention were calculated independently. The primary outcome was variations in insulin resistance, which was assessed by proxy as the difference between HOMA-IR scores at the beginning and end of the trials. The results suggested that LoGI diets decrease HOMA-IR scores to a greater extent than HiGI diets (estimate: 0.31; 95% confidence interval [CI], 0.01–0.61; *p* < 0.001) in individuals without diabetes mellitus. This association remained significant (estimate: 0.16; 95% CI, 0.01–0.31) after excluding one study with a short follow-up time (7 days). In conclusion, LoGI diets lower HOMA-IR scores to a greater extent than HiGI diets in adults without diabetes mellitus.

**Systematic review registration:**

(https://www.crd.york.ac.uk/PROSPERO/).

## Background

Type 2 diabetes mellitus is a serious and increasingly prevalent health problem. In 2019, 463 million people worldwide (9.3% of the global population) had type 2 diabetes, an increase from 153 million in 1980. The estimated worldwide prevalence of impaired glucose intolerance is approximately 7.5% and is expected to increase to 8% by 2030 (1). More than one million deaths annually are attributable to type 2 diabetes mellitus, rendering it the ninth leading cause of mortality worldwide (2), and the World Health Organization anticipates that diabetes mellitus will become the seventh leading cause of death by 2030 (3). Numerous randomized controlled trials have demonstrated that adopting a healthy dietary pattern and making lifestyle modifications is effective in preventing type 2 diabetes mellitus. As indicated by a previous meta-analysis, lifestyle interventions aimed at weight reduction, increased physical activity, or dietary modification decreased the risk of developing type 2 diabetes mellitus by 47% (4).

In 1981, Jenkins and colleagues introduced the Glycemic Index to quantify the relative impact of carbohydrates on postprandial blood glucose levels, using glucose or white bread as the reference standard (5). The glycemic index is a ranking scale for foods that range from 0 to 100 on the basis of this influence and classifies them into three groups: foods with a low glycemic index (LoGI) score 55 of or less, those with a moderate glycemic index score between 56 and 69, and those with a high glycemic index (HiGI) score of 70 or greater. LoGI foods result in a low post-meal blood sugar spike, whereas HiGI foods cause a high blood sugar spike (6). LoGI diets are beneficial for people with diabetes because they regulate blood sugar levels and prevent blood sugar spikes. Wang and colleagues’ systematic review suggested that LoGI diets promote blood sugar control in individuals with diabetes (7). Similarly, Ojo and colleagues conducted a meta-analysis and discovered that LoGI diets improved levels of glycated hemoglobin (HbA1c) and fasting blood sugar compared with HiGI diets in patients with type 2 diabetes (8). In addition, a systematic review and dose–response meta-analysis found that a 5-unit increase in GI is associated with an 8% increased risk of developing type 2 diabetes mellitus (9). Furthermore, a meta-analysis of prospective cohort studies indicated that for every 10-unit increase in GI, the relative risk of type 2 diabetes mellitus rises by 27% (10).

The homeostasis model assessment of insulin resistance (HOMA-IR) was introduced in 1985 and is used to quantify insulin resistance (IR) and beta-cell function using basal glucose and insulin concentrations. The HOMA-IR measures the effects of the physiological feedback loop resulting from attenuated insulin suppression during hepatic glucose production (11). Results from the HOMA-IR model have been found to be strongly correlated with euglycemic clamp results (12, 13). Several studies have revealed the positive effects of LoGI diets on insulin sensitivity and other metabolic parameters (14–17). LoGI diets with carbohydrates and fiber help stabilize blood glucose levels, reduce insulin resistance, and lower risk factors for cardiovascular diseases. These diets enable rapid weight loss, reduce fasting glucose and insulin levels, lower circulating triglycerides, and improve blood pressure (16, 17). The benefits of LoGI diets in reducing hyperinsulinemia, dyslipidemia, and inflammatory markers to reduce cardiovascular risk have also been examined (16).

To date, observational studies based on single cohorts have provided contradictory results regarding whether a LoGI diet can lower IR in adults without diabetes mellitus with no comorbidities. Du et al. conducted a cross-sectional analysis of two population-based cohort studies (the CoDAM Study and the Hoorn Study), including 974 participants, and demonstrated that dietary GI levels had a substantial association with fasting insulin levels and HOMA-IR; every 10 units of insulin increase in GI was associated with a 23% increase in IR (18). Additionally, McClenaghan observed that dietary GI levels were significantly and positively associated with fasting insulin levels in the 2,941 participants in the Framingham Offspring study (19). However, another study obtained opposite results (20), and several studies have revealed no significant effects of a LoGI diet on IR (21, 22). These inconsistencies might be attributed to several factors, including variability in study designs, differences in participant populations (e.g., inclusion of individuals with diabetes or other comorbidities), and the use of diverse methods to assess IR.

We conducted the present systematic review to evaluate the effects of LoGI diets on IR. In contrast to other studies, our review focused on healthy adults without diabetes mellitus or other comorbidities and explored the associations between various GI diets and a reduction in IR, a critical factor in the pathogenesis of type 2 diabetes mellitus. Due to the high heterogeneity and various measurements of IR, we included RCTs that compared clearly defined LoGI diets with HiGI diets and used HOMA-IR as an indicator of IR because it is reliable, minimally invasive, and widely used in assessing patients with prediabetes.

## Methods

### Protocol and registration

This systematic review and meta-analysis followed the Preferred Reporting Items for Systematic Reviews and Meta-Analyses (PRISMA) guidelines (23), detailed in the Supplementary material, and is registered with PROSPERO under CRD42023478554 (https://www.crd.york.ac.uk/PROSPERO/).

### Eligibility criteria and search strategy

This meta-analysis systematically searched the literature to identify RCTs comparing LoGI diets with HiGI diets in individuals without diabetes mellitus or other substantial comorbidities. LoGI and HiGI groups were determined from the design of the included studies. We comprehensively searched for all articles published between 2000 and 2025 on PubMed,^1^ Embase,^2^ Cochrane Library,^3^ and Clinical Trials registration.^4^ For example, PubMed was searched using the medical subheadings “Glycemic Index,” “Insulin Resistance,” and restricted article type “Randomized Controlled Trial” in humans. RCTs were included if they were written in English and met the following inclusion criteria: (1) using a LoGI diet as the main component of the intervention arm and a HiGI diet in the control arm only; (2) being noncrossover RCTs with clearly defined LoGI and HiGI diets in the study protocol; (3) having study participants >18 years old who had no severe systemic comorbidities such as diabetes mellitus, coronary artery disease, or end-stage renal disease, who were not undergoing major surgery in the 6 months preceding the trial, who had no other systemic diseases requiring hospital admission, and who did not receive oral hypoglycemic agents or insulin treatment; and (4) having recorded data on insulin sensitivity and HOMA-IR before and after the dietary interventions. The detailed research policy is provided in the Supplementary material.

The titles and abstracts of all articles were used to determine relevance. Full articles were screened if the titles and abstracts were relevant to the objectives of the present study. Two reviewers (YTY and YHF) conducted searches independently and combined their findings.

### Data extraction and items

The following information was extracted from each article: the author’s name, publication year, study design, trial country, trial duration, dietary intervention, participant count, average age, and diet composition. Two independent reviewers (YTY and YHF) independently extracted data from and assessed methodological quality in the evaluated trials. Disagreements were settled through discussions between the researchers to achieve consensus.

The outcome measure of HOMA-IR quantifies insulin homeostasis using a simple equation in which the insulin–glucose product is divided by a constant. The equation, originally proposed by Andrade et al. (24), is as follows: IR_HOMA_ = [insulin level (μU/mL) × glucose level (mmol/L)]/22.5. This index is particularly useful in large epidemiological studies when only fasting insulin and glucose values are available. The use of HOMA-IR to assess IR was also validated in a study of adolescents.

Key study variables were also extracted, and they included participant demographics (number, gender ratio, mean age), dietary interventions (glycemic index, macronutrient composition, fiber content), and dietary controls (diet provision method, recording approach). Intervention details covered diet type (iso- or hypocaloric) and exercise involvement. Study designs tracked intervention duration (7 days to 6 months), location, and year. These data were consistently measured across both LoGI and HiGI groups for comparative analysis.

### Assessment of study quality

The Risk of Bias (ROB) evaluation in this study was conducted using the Cochrane Risk of Bias Tool (25), which assesses methodological quality across domains such as randomization, allocation concealment, blinding, incomplete outcome data, and selective reporting. Two independent reviewers (YTY and YHF) evaluated all studies. Any discrepancies were resolved by discussions between the two investigators until a consensus was reached.

### Data synthesis and statistical analysis

Data extraction and analysis were conducted using the techniques outlined in the *Cochrane Handbook for Systematic Reviews of Interventions* (Version 6.2, 2021) (25). The primary outcomes of our study were HOMA-IR values before and after the dietary interventions. Subgroup analysis was conducted to segment study populations according to follow-up durations and methods used to provide diets with specific GIs to the participants. Heterogeneity between studies was assessed by reviewing trial data, and statistical heterogeneity was analyzed using a chi-squared test. The *I*^2^ statistic was calculated to assess inconsistency between studies, and an *I*^2^ value >50% was considered to indicate substantial heterogeneity (26). A random effects model was employed in our analysis to accommodate this heterogeneity.

We treated the outcome of HOMA-IR as a continuous variable and calculated standardized mean differences (SMD) with 95% confidence intervals (CIs). Standard deviations were used directly if reported by the author or calculated from the observed mean differences and test statistics. In cases where the test statistics were unavailable, we estimated the corresponding *p* values using normal distribution tables based on the test statistics provided. Potential publication bias was assessed through the use of funnel plots, and this analysis was further supported by applying Egger’s test to evaluate asymmetry. Egger’s test quantitatively evaluates the asymmetry of the funnel plot by testing for a linear relationship between the effect size and the standard error. Sensitive analysis explored heterogeneity by excluding shorter follow-up studies, while subgroup analyses evaluated the robustness of results by examining dietary provision methods and supervised exercise.

Statistical analyses were conducted using Stata version 15 (StataCorp, College Station, TX, United States). Two-tailed *p* values < 0.05 were considered statistically significant.

## Results

### Selection of studies and study description

**Figure 1:**
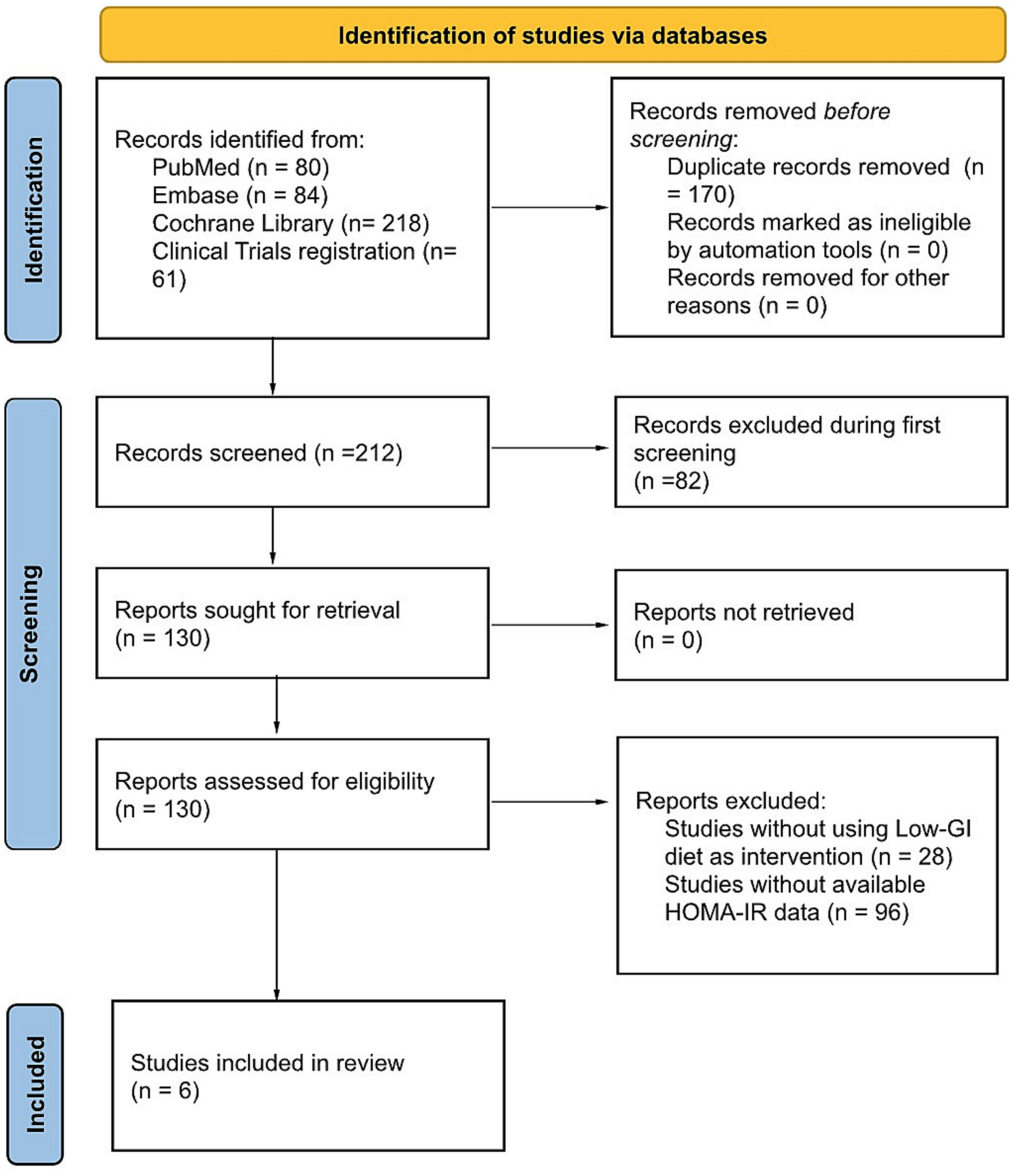
PRISMA flow diagram of the study selection. GI: glycemic index; HOMA-IR, homeostatic model assessment for insulin resistance.

The PRISMA flowchart for the review process and the selection of the RCTs included in our analysis is depicted in Figure 1. A total of 212 studies were returned using our search terms. Of these, 130 were identified as RCTs and were screened for relevance. Finally, six studies involving 192 participants met our selection criteria and were included in our meta-analysis (27–32). The characteristics of the studies included in the meta-analysis are presented in Table 1. The 192 participants were divided into two groups: 100 participants who received a LoGI diet and 92 participants who received a HiGI diet.

**Table 1:** Characteristics of included studies.

Authors of study	Year	Location	Duration	Other intervention	LoGI diet participants				HiGI diet participants			
					Number of cases (male/female)	Mean age (year)	GI index (AU)	Characteristics of diets	Number of cases (male/female)	Mean age (year)	GI index (AU)	Characteristics of diets
Pittas et al. (31)	2006	United States	6 months	Non	16 (3/13)	34.3	53	Energy from carbohydrate: 40% of kcal Energy from protein: 30% of kcal Energy from fat: 30% of kcal Fiber: 15 g/1,000 kcal	16 (4/12)	35.0	86	Energy from carbohydrate: 60% of kcal Energy from protein: 20% of kcal Energy from fat:20% of kcal Fiber: 15 g/1,000 kcal
Haus et al. (27)	2011	United States	7 days	Supervised aerobic exercise	7 (3/4)	68	41.1	Carbohydrate: 252.6 ± 13.1 g/day Protein: 79.4 ± 4.0 g/day Fat: 56.6 ± 2.9 g/day Fiber: 28.8 ± 1.6 g/day	8 (3/5)	61	80.9	Carbohydrate: 222 ± 78 g/day Protein: 90 ± 32 g/day Fat: 68 ± 32 g/day Fiber: 21 ± 6 g/day
Armendáriz-Anguiano et al. (30)	2011	Mexico	6 months	Non	16 (n/a)	36.9	52	Carbohydrate: 222 ± 78 g/day Protein: 90 ± 32 g/day Fat: 68 ± 32 g/day Fiber: 21 ± 6 g/day	8 (n/a)*	33.8	57	Carbohydrate: 270 ± 92/g day Protein: 80 ± 19 g/day Fat: 65 ± 28 g/day Fiber: 19 ± 13 g/day
Malin et al. (29)	2012	United States	4 months	Supervised aerobic exercise	11 (4/7)	67.2	40.3	Carbohydrate: 256.7 ± 11.8 g/day Protein: 58.2 ± 2.3 g/day Fat: 78.9 ± 3.5 g/day Fiber: 29.5 ± 1.3 g/day	10 (7/3)	65.6	80.3	Carbohydrate: 301.0 ± 20.2 g/day Protein: 66.3 ± 3.9 g/day Fat: 88.6 ± 5.6 g/day Fiber: 30.5 ± 2.1 g/day
Juanola-Falgarona et al. (28)	2014	Spain	6 months	Non	41 (8/33)	42.5	34	Energy from carbohydrate: 42% of kcal Energy from protein: 18% of kcal Energy from fat: 40% of kcal	40 (7/33)	44.0	62	Energy from carbohydrates: 42% of kcal Energy from protein: 18% of kcal Energy from total fat: 40% of kcal
Mulya et al. (32)	2017	United States	4 months	Supervised aerobic exercise	9 (4/5)	67	40	Energy from carbohydrate: 51.7% of kcal Energy from fat: 48.3% of kcal	10 (6/4)	64	80	Energy from carbohydrate: 51.6% of kcal Energy from fat: 48.4% of kcal

LoGI, Low glycemic index; HiGI, high glycemic index; GI, glycemic index; kcal, kilocalorie.

### Characteristics of included studies

The duration of the included studies ranged from 1 week to 6 months. The mean age of the participants ranged from 34.3 to 68 years. The characteristics of the LoGI/HiGI dietary interventions included in the present meta-analysis are presented in Table 2. Of the included RCTs, four involved intervention diets prepared or recorded by the researchers, whereas two studies used diets suggested by a dietitian and prepared by the participants themselves (28, 30). Three trials involved supervised aerobic exercises such as treadmill walking (27, 29, 32). Moreover, two RCTs (28, 31) employed hypocaloric meals, and three employed isocaloric meals (27, 29, 32).

**Table 2:** Diet control design of the studies.

Authors of study	Diet intervention	Diet recording	Daily total caloric consumption	Exercise intervention
Pittas et al. (31)	Provisioned by study group	Recorded by study group	Hypocaloric	Non
Haus et al. (27)	Provisioned by study group	Recorded by study group	Iso-caloric	Supervised aerobic exercise
Armendáriz-Anguiano et al. (30)	Advised by dietitian	Self-recorded	Not controlled	Non
Malin et al. (29)	Provisioned by study group	Recorded by study group	Iso-caloric	Supervised aerobic exercise
Juanola-Falgarona et al. (28)	Advised by dietitian	Self-recorded	Hypocaloric	Non
Mulya et al. (32)	Provisioned by study group	Recorded by study group	Iso-caloric	Supervised aerobic exercise

### Settings

The studies included in our analysis were conducted in the United States, Mexico, or Spain.

### Quality of trials

**Figure 2:**
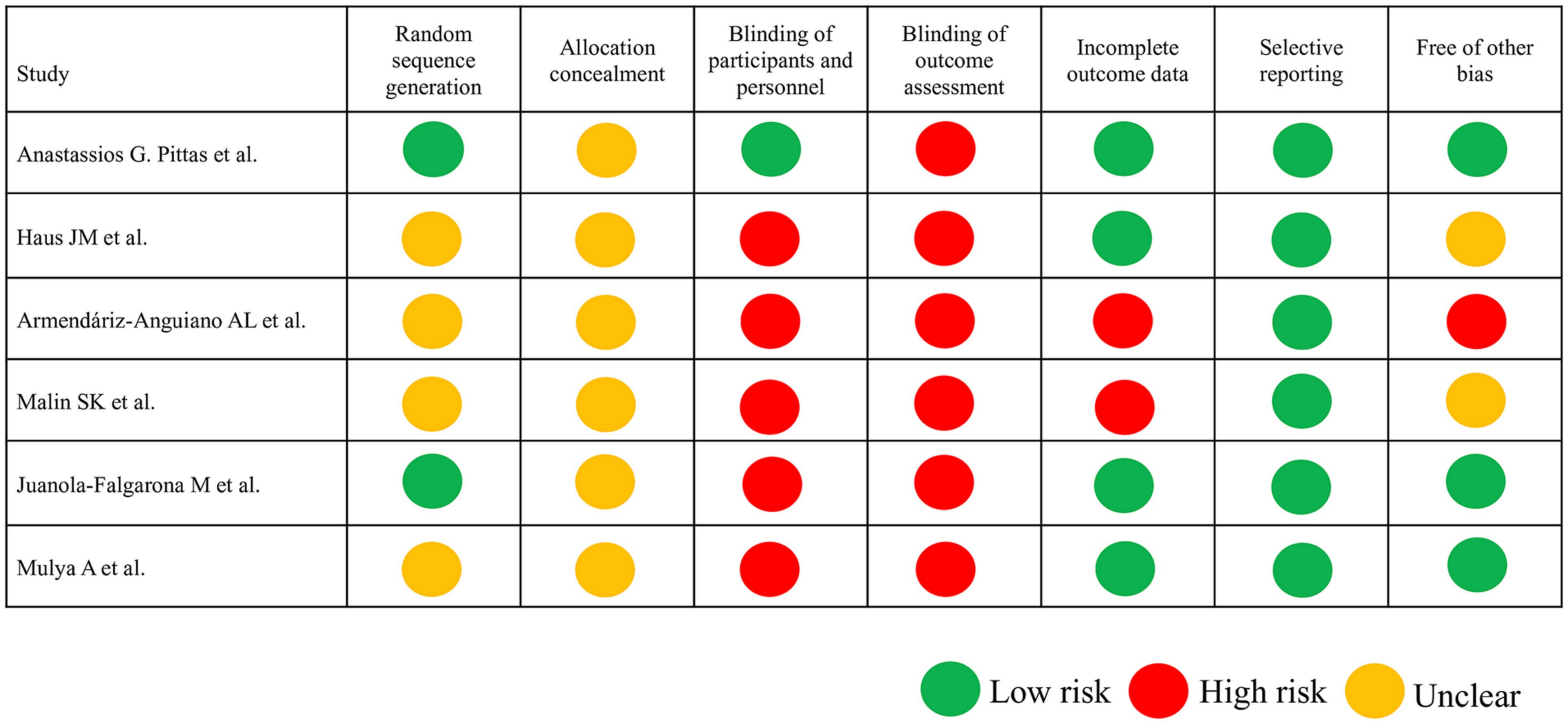
Risk of bias assessment according to the Cochrane guidelines. In the six included trials, low risk of bias is indicated by a green circle, unclear risk of bias is indicated by a yellow circle, and high risk of bias is indicated by a red circle.

The ROB assessment revealed varied methodological quality among studies (Figure 2). Because the intervention in the RCTs was the type of diet administered, establishing a double-blind protocol was not usually possible. Random sequence generation was explicitly reported in two studies (28, 31), while others lacked sufficient details, leading to an unclear risk of bias in this domain. Double-blinding was not feasible in most dietary intervention studies, with only one study (31) attempting blinding of outcomes, resulting in high performance and detection bias risks. The lack of outcome assessment blinding in the studies led to significant performance and detection bias, particularly for subjective outcomes like self-reported dietary adherence. Selective reporting is uniformly rated as low risk, showcasing consistency in this parameter.

### Effects of LoGI and HiGI diets on HOMA-IR in adults without diabetes mellitus

**Figure 3:**
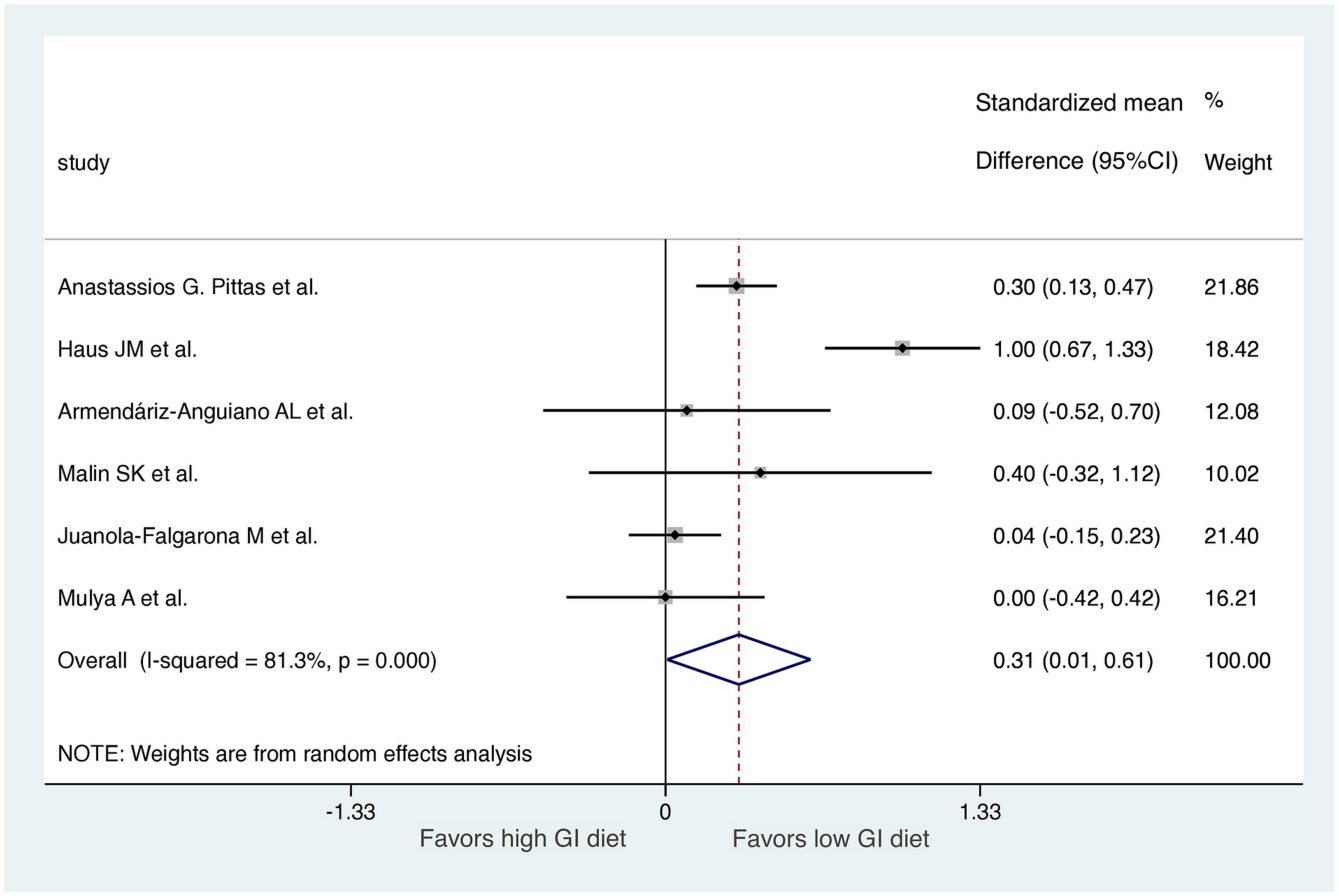
Forest plot of standardized mean differences in HOMA-IR scores between high GI and low-GI diets in the six included randomized controlled trials. For each study, the shaded square indicates the estimated effect of the intervention. A horizontal line connects the lowest and highest points of the 95% CIs for these effects. The size of the shaded square indicates the influence of the study in the overall meta-analysis. At the bottom of the graph, the diamond represents the combined weighted standardized mean difference of HOMA-IR and the 95% CI for the five included studies. CI, confidence interval; GI, glycemic index; HOMA-IR, homeostatic model assessment for insulin resistance.

**Figure 4:**
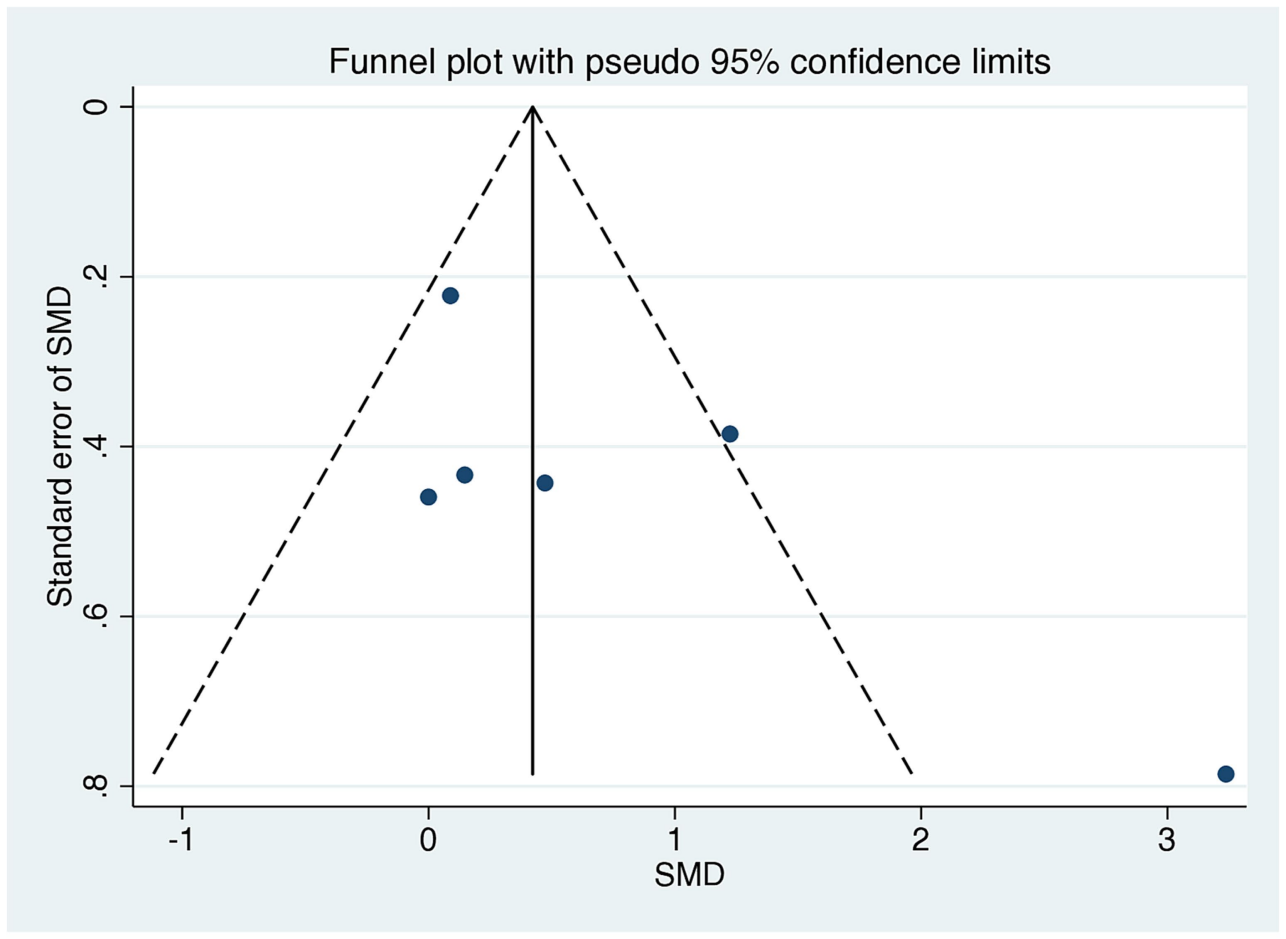
Funnel plots evaluating publication bias in studies comparing HOMA-IR levels in low GI and high GI diets. Egger’s test revealed no significant publication bias (*p* = 0.140). SMD, standardized mean difference; CI, confidence interval; GI, glycemic index; HOMA-IR, homeostatic model assessment for insulin resistance.

A comparison of HOMA-IR values before and after dietary interventions revealed that two of the six RCTs demonstrated a substantial decrease in IR in response to the LoGI diet compared with the HiGI diet (27, 31). Four of the six RCTs observed no significant difference in HOMA-IR between the LoGI and HiGI arms (28–30, 32) (Figure 3). However, the results of the overall meta-analysis with a random effect suggested that the LoGI diet resulted in a substantially lower IR than the HiGI diet (SMD, 0.31; 95% CI, 0.01–0.61). Substantial heterogeneity was observed between the trials (*I*^2^ = 81.3%, *p* < 0.001), and the funnel plot (Egger’s test) indicated no significant evidence of small-study effects or publication bias (*p* = 0.092; Figure 4).

### Sensitivity and subgroup analysis

**Figure 5:**
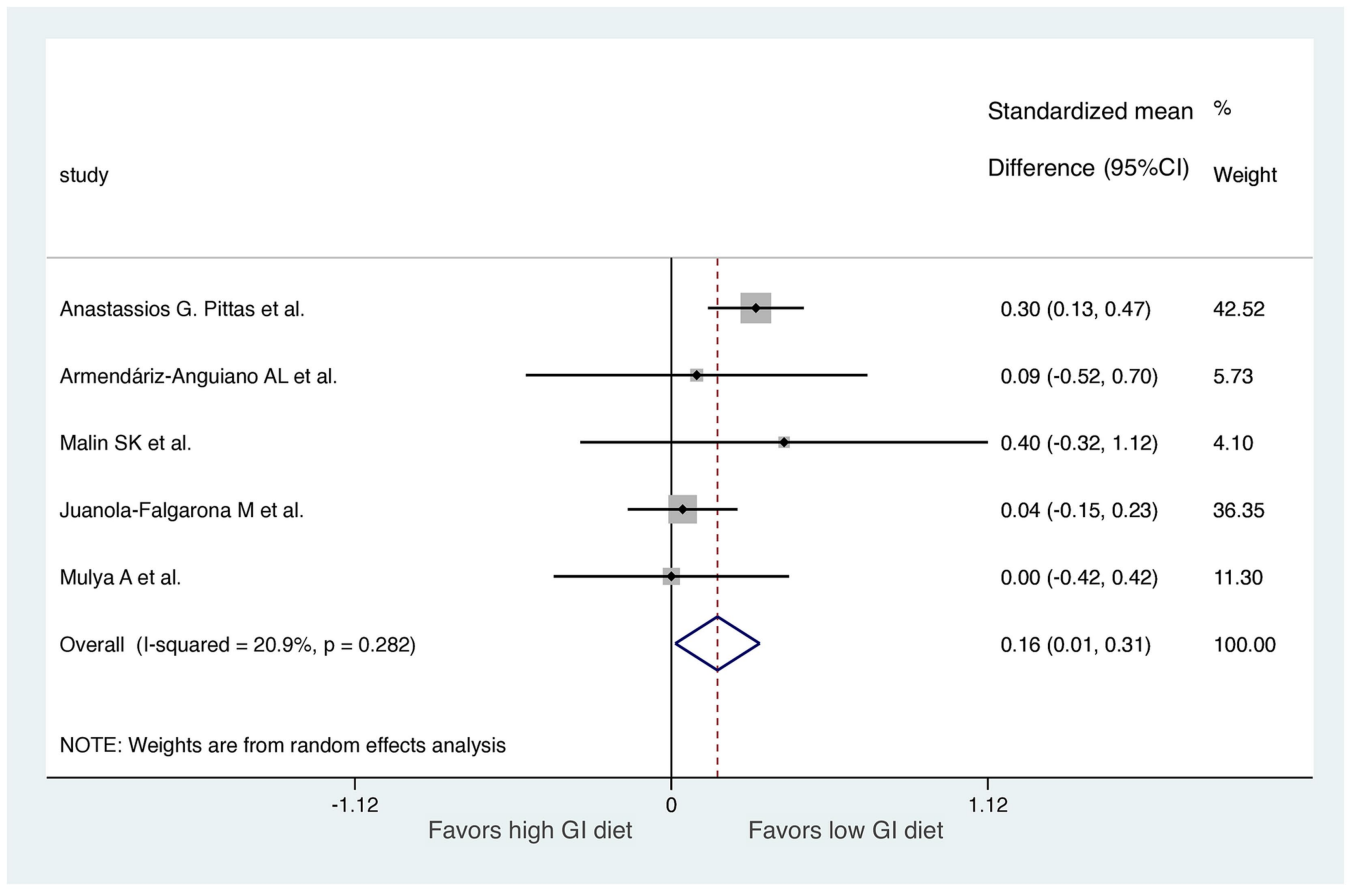
Sensitive analysis excluding the study with a follow-up time of 1 week. For each study, the shaded square indicates the estimated effect of the intervention. A horizontal line connects the lowest and highest points of the 95% CIs for these effects. The size of the shaded square indicates the influence of the study in the overall meta-analysis. At the bottom of the graph, the diamond represents the combined weighted standardized mean difference of HOMA-IR and the 95% CI for the five included studies. CI, confidence interval; GI, glycemic index; HOMA-IR, homeostatic model assessment for insulin resistance.

Studies were segmented by follow-up duration in a sensitive analysis. We excluded one study with only a 7-day follow-up (27). The results of the meta-analysis revealed a significant difference in HOMA-IR score improvement between the LoGI and HiGI diets (mean difference, 0.16; 95% CI, 0.01–0.31; Figure 5). Moreover, the sensitive analysis by excluding a 7-day follow up study reduced heterogeneity (*I*^2^ = 81.3 to 20.9%), indicating that this study was the primary contributor to the heterogeneity.

**Figure 6:**
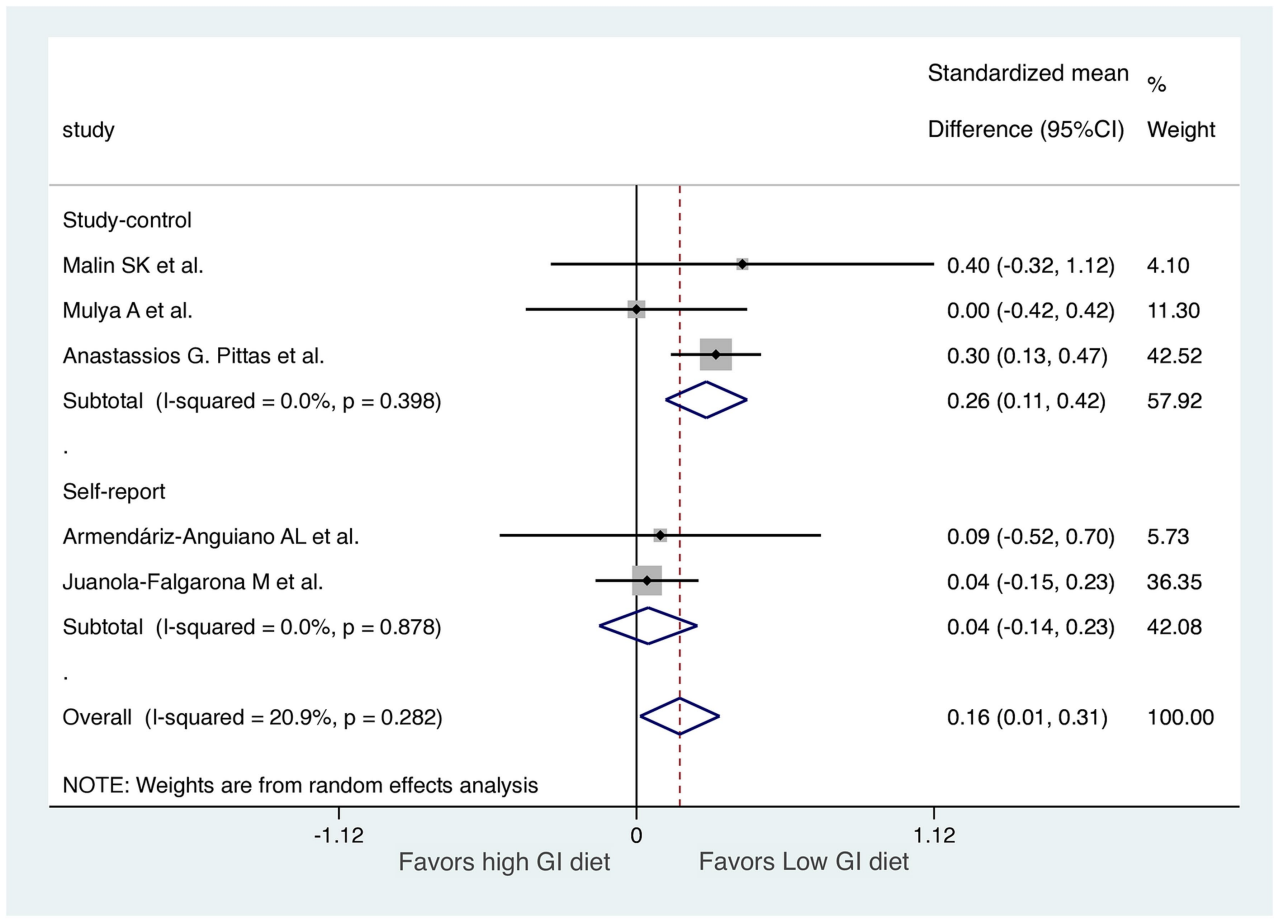
Subgroup analysis by method recording the diet (study control vs. self-report). For each study, the shaded square indicates the estimated effect of the intervention. A horizontal line connects the lowest and highest points of the 95% CIs for these effects. The size of the shaded square indicates the influence of the study in the overall meta-analysis. At the bottom of the graph, the diamond represents the combined weighted standardized mean difference of HOMA-IR and the 95% CI for the five included studies. CI, confidence interval; GI, glycemic index; HOMA-IR, homeostatic model assessment for insulin resistance.

**Figure 7:**
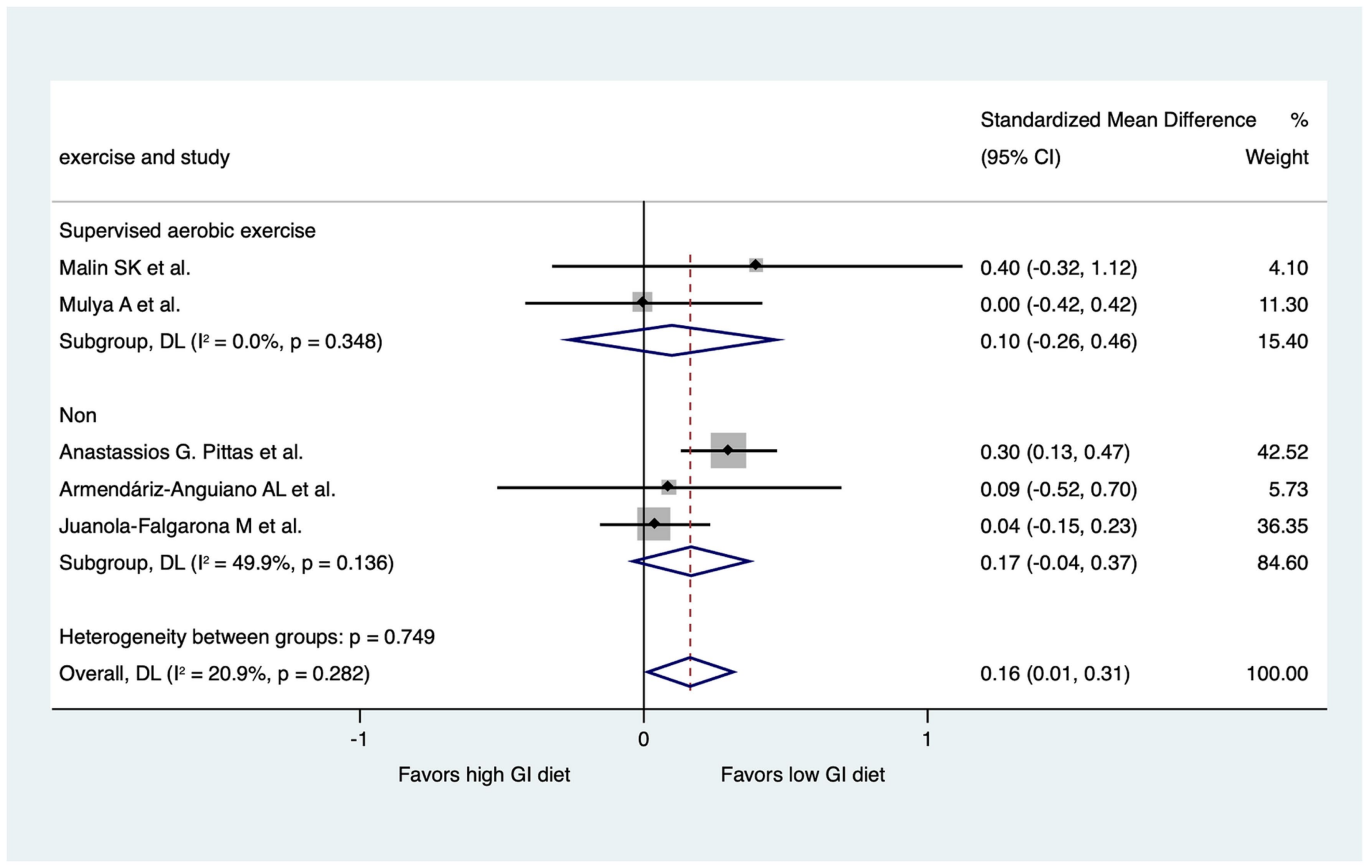
Subgroup analysis by the presence of supervised aerobic exercise. For each study, the shaded square indicates the estimated effect of the intervention. A horizontal line connects the lowest and highest points of the 95% CIs for these effects. The size of the shaded square indicates the influence of the study in the overall meta-analysis. At the bottom of the graph, the diamond represents the combined weighted standardized mean difference of HOMA-IR and the 95% CI for the five included studies. CI, confidence interval; GI, glycemic index; HOMA-IR, homeostatic model assessment for insulin resistance.

We performed two subgroup analyses to assess diet GI methods and exercise types in the RCTs, excluding the 7-day follow-up study (Figures 6, 7). RCTs that provided diets directly from a hospital (31) or the study group (29, 32) were classified into a “study control” group. RCTs that required participants to prepare their own diets according to individualized menus (30) or following the advice of a dietitian (28) were classified as a “self-report” group. The results revealed significant improvements in HOMA-IR (LoGI vs. HiGI diets) in the study control group (SMD, 0.26; 95% CI 0.11–0.42) but not in the self-report group (SMD, 0.04; 95% CI −0.14 to 0.23) (Figure 6). In the supervised aerobic exercise group (29, 32), the LoGI diet did not significantly improve in HOMA-IR (SMD, 0.10; 95% CI, −0.26 to 0.46). Similarly, studies without supervised exercise (28, 30, 31) also showed no significant improvement (SMD, 0.17; 95% CI, −0.04 to 0.37). However, there is a trend favoring the LOGI diet (Figure 7).

## Discussion

Our meta-analysis of noncrossover RCTs revealed that LoGI diet groups had substantially lower IR than HiGI diet groups in the population without diabetes mellitus. To our knowledge, this is the first meta-analysis to focus on the effects of LoGI diets and HiGI diets with clearly defined GIs on IR in adults without diabetes mellitus with comparable IR levels (assessed using HOMA-IR). Our findings indicate that LoGI diets may benefit individuals without diabetes mellitus. This study underscores the need to incorporate LoGI dietary recommendations into preventive healthcare practices and public health policies focused on diabetes prevention.

The clinical significance of the findings in this study lies in the demonstrated benefits of a LoGI diet in improving IR in adults without diabetes mellitus. While the SMD of 0.31 (95% CI: 0.01–0.61) suggests a small to moderate effect size (33), this improvement is clinically meaningful. Even modest reductions in HOMA-IR can contribute to better insulin sensitivity, potentially reducing the risk of developing type 2 diabetes mellitus over time (34, 35). Reducing the incidence of type 2 diabetes will lead to significant improvements in cardiovascular health by lowering the risks associated with heart disease and related complications (36).

A LoGI diet, which has proven benefits for individuals with type 2 diabetes mellitus, also benefits healthy individuals (37). LoGI diets enhance IR through several mechanisms. They help stabilize blood sugar and insulin levels by reducing spikes in blood glucose and insulin that typically occur after consuming meals (14). This stabilization persists throughout the day, providing a more consistent metabolic environment, which not only lowers the risk of heart disease but also improves the insulin sensitivity over time. In contrast, individuals who follow HiGI diets tend to experience elevated levels of circulating free fatty acids, which can interfere with proper insulin signaling. Consequently, these increased fatty acid levels contribute to the accumulation of fat in various tissues of liver and muscles. This buildup of fat further disrupts metabolic processes, ultimately leading to an increased risk of IR (38). In comparison, a LoGI diet lowers levels of these fatty acids, improving insulin resistance over time. Furthermore, one meta-analysis (39) uncovered an association of LoGI diets with weight loss. This association is significant because weight loss plays an important role in improving overall health and reducing factors that contribute to cardiovascular diseases (40). By promoting healthier weight management, LoGI diets can help lower the risk of heart-related conditions and improve metabolic outcomes. Finally, Finally, LoGI diets may have an impact on gut hormones, including glucose-dependent insulinotropic polypeptides, which play a role in regulating important metabolic processes. These hormones are involved in promoting insulin secretion in response to food intake, helping the body effectively manage blood glucose levels. By influencing the activity of these gut hormones, LoGI diets can support better glucose regulation and contribute to improved insulin dynamics over time, enhancing overall metabolic control (41).

Shikany et al. conducted a crossover RCT involving 24 obese patients without diabetes mellitus and demonstrated that fasting glucose and insulin decreased in the LoGI diet group compared with the HiGI diet group, although this decrease was not statistically significant (42). However, Gao et al. revealed a substantial decrease in IR in a single-center crossover study that compared a LoGI diet with a normal GI hospital diet (43). These results suggest that in diets with a high carbohydrate content, those with a LoGI decreased insulin sensitivity significantly (−20%, *p* = 0.002) compared with HiGI diets, a benefit not observed in diets with a low carbohydrate content. Crossover study design has frequently been used in nutritional intervention trials; however, to avoid a “carryover effect” due to a lack of uniformity in the duration of the LoGI/HiGI follow-up time, we selected RCTs without crossover designs (44).

Although a previous study demonstrated that IR is dynamic and can be altered in as few as 3 days after dietary and lifestyle changes (45), we excluded one study that had a short observation period (7 days) to avoid the bias of insufficient follow-up time (27). After excluding this study, relative to HiGI diets, LoGI diets became more beneficial in terms of changes in HOMA-IR in adults without diabetes mellitus. Our results also revealed that the RCTs with diets prepared by the researchers or the study group exhibited a substantial difference in HOMA-IR between LoGI and HiGI diets not observed in studies with diets prepared and reported by the participants themselves. This variation in HOMA-IR between food preparation methods may be due to a recall bias induced by the self-prepared and self-reported methodologies.

Regular supervised aerobic exercise, often combined with resistance training, shows consistent improvements in glucose metabolism, reducing HOMA-IR in those with metabolic syndrome (46). However, in our subgroup analysis, we did not observe the significant improvement in HOMA-IR within the supervised aerobic exercise group. This lack of improvement is likely attributable to several factors, including a small sample size and the considerable variability in the exercise protocols employed across the studies. Therefore, further study is needed to clarified it.

The substantial heterogeneity observed in the initial meta-analysis (*I*^2^ = 81.3%,) was significantly reduced to 20.9% after excluding a study with short follow-up durations, specifically the study with a 7-day follow-up (as shown in Figures 3, 5). This indicates that the short follow-up period was a major contributor to the heterogeneity in this meta-analysis. Short follow-up durations may not allow sufficient time for dietary interventions to produce measurable changes in insulin resistance, leading to variability in the results. In our analysis, the significant association continued to hold even after the exclusion of the study with a 7-day follow-up period. This exclusion resulted in a notable reduction in heterogeneity, suggesting that the findings became more consistent and less influenced by variability between studies.

Our study has several strengths. First, we only included RCTs with clearly defined LoGI/HiGI dietary interventions, which can be more easily interpreted than observational cohort studies. Second, we conducted a subgroup analysis to determine whether dietary source had a significant effect on the results of the meta-analysis in the event that the self-reported design introduced a recall bias. Third, we used the random-effects model to obtain accurate results despite substantial heterogeneity among studies (*I*^2^ = 81.3%). This model accounts for variations in true effect sizes across studies, considering both within-study and between-study differences, making it suitable for diverse study characteristics. In contrast, a fixed-effects model assumes a single true effect size, ignoring between-study variability, which could narrow confidence intervals, underestimate uncertainty, and overstate precision. Thus, the random-effects model ensured more reliable conclusions.

Despite these strengths, our study has some limitations. First, our study included studies employing diets with various caloric intakes; therefore, the effects of dietary glucose loading independent of caloric restriction could not be assessed. Various studies have revealed that caloric and other macronutrient components are individually associated with metabolic parameters (47, 48). However, because we only included RCTs, this limitation is unlikely to have prejudiced our results. Second, excluding crossover RCTs to avoid the carryover effect may have led to the omission of relevant data that could contribute to understanding the short-term dynamics of IR changes in response to dietary interventions. Finally, The small sample sizes in the included RCTs may reduce the power and reliability of the meta-analysis, increasing the risk of Type II errors, imprecise effect size estimates, wider confidence intervals, and greater susceptibility to heterogeneity and publication bias. Larger, well-designed studies across diverse regions are needed to clarify the effects of varying GI diets on insulin resistance in healthy individuals. Finally, the sample sizes of the included RCTs were small, and additional large-scale studies in diverse regions are required to elucidate the effects of varying GI diets on the IR of healthy individuals.

## Conclusion

This meta-analysis based on Cochrane methodology revealed that a LoGI diet was associated with a significantly lower HOMA-IR than a HiGI diet in adults without diabetes mellitus. This finding indicates that early intervention with a LoGI diet can reduce the risk of developing diabetes mellitus by decreasing IR.

## Data Availability

The original contributions presented in the study are included in the article/Supplementary material, further inquiries can be directed to the corresponding author.

## Footnotes

^1^https://pubmed.ncbi.nlm.nih.gov/; accessed 01 February 2025.

^2^https://www.embase.com; accessed 01 February 2025.

^3^https://www.cochranelibrary.com; accessed 01 February 2025.

^4^https://clinicaltrials.gov/; accessed 01 February 2025.
