# Supplementary material for: Effect of dietary glycemic index on insulin resistance in adults without diabetes mellitus: a systematic review and meta-analysis
Source: Front Nutr. 2025 Feb 13;12:1458353. doi: 10.3389/fnut.2025.1458353 (PMC11864931; doi:10.3389/fnut.2025.1458353)
Supplement: Supplementary file 2 [file Table_2.docx]

**Supplement table 2 . Complete Search Strategy**

**Databases and Search Dates**

- We searched **PubMed (MEDLINE)**, **Embase**, the **Cochrane Library (CENTRAL)**, and **ClinicalTrials.gov** on **1 January 2025**.
- We included articles published (or trials registered) between **2000 and 2024**.

**1. PubMed (MEDLINE)**

- **URL**: <https://pubmed.ncbi.nlm.nih.gov/>
- **Date Accessed**: 1 January 2025
- **Limits**: Publication date from 1 January 2000 to 31 December 2024; Humans; Adults; Randomized Controlled Trials; excluding diabetes-related studies

**Search Terms (MeSH-Focused)**
(
“Glycemic Index”[Mesh]
AND (“Diet”[Mesh] OR “Diet Therapy”[Mesh])
AND “Insulin Resistance”[Mesh]
AND “Randomized Controlled Trials as Topic”[Mesh]
AND “Humans”[Mesh]
AND “Adult”[Mesh]
NOT (“Diabetes Mellitus”[Mesh] OR “Diabetes Mellitus, Type 1”[Mesh] OR “Diabetes Mellitus, Type 2”[Mesh] OR “Diabetes, Gestational”[Mesh])
)
AND (“2000/01/01”[PDAT] : “2024/12/31”[PDAT])

**2. Embase**

- **URL**: <https://www.embase.com/>
- **Date Accessed**: 1 January 2025
- **Limits**: Publication years from 2000 to 2024; Human; Adult; Randomized Controlled Trial; excluding diabetes-related studies

**Search Terms (Emtree-Focused)**
(
‘glycemic index’/exp
AND (‘diet’/exp OR ‘diet therapy’/exp)
AND ‘insulin resistance’/exp
AND ‘randomized controlled trial’/exp
AND ‘human’/exp
AND ‘adult’/exp
NOT (‘diabetes mellitus’/exp OR ‘diabetes mellitus type 1’/exp OR ‘diabetes mellitus type 2’/exp OR ‘gestational diabetes’/exp)
)
AND [2000-2024]/py

**3. Cochrane Library (CENTRAL)**

- **URL**: <https://www.cochranelibrary.com/>
- **Date Accessed**: 1 January 2025
- **Limits**: Year of Publication 2000–2024; Randomized Controlled Trials; excluding diabetes-related studies; adult human populations

**Search Approach (in Search Manager)**
Line 1: MESH descriptor: “Glycemic Index”
Line 2: MESH descriptor: “Diet” OR MESH descriptor: “Diet Therapy”
Line 3: MESH descriptor: “Insulin Resistance”
Line 4: MESH descriptor: “Randomized Controlled Trials as Topic”
Line 5: MESH descriptor: “Humans”
Line 6: MESH descriptor: “Adult”
Line 7: MESH descriptor: “Diabetes Mellitus” OR MESH descriptor: “Diabetes Mellitus, Type 1” OR MESH descriptor: “Diabetes Mellitus, Type 2” OR MESH descriptor: “Diabetes, Gestational”

Final combination:
Line 8: #1 AND #2 AND #3 AND #4 AND #5 AND #6 NOT #7

We then applied the filter for Year of Publication (2000–2024) in the Cochrane Library interface.

**4. ClinicalTrials.gov**

- **URL**: <https://clinicaltrials.gov/>
- **Date Accessed**: 1 January 2025
- **Limits**: Interventional (Clinical Trial); Adult participants; First Posted between 2000 and 2024

**Search Terms (Advanced Search)**
glycemic index
AND (insulin resistance OR insulin sensitivity)
AND (diet OR dietary)
AND randomized
NOT diabetes

We selected “Interventional (Clinical Trial)” under Study Type and limited the Age Group to “Adult,” specifying the date range for First Posted between 1 January 2000 and 31 December 2024.

**Screening and Eligibility**

1. **Initial Screening**
   - Two independent reviewers examined titles and abstracts of all retrieved records, removed duplicates, and excluded records unrelated to the effect of dietary glycemic index on insulin resistance in adults without diabetes mellitus.
2. **Full-Text Review**
   - We obtained full texts of studies or read full ClinicalTrials.gov entries that appeared to meet our inclusion criteria: (i) randomized controlled trials, (ii) participants aged ≥18 years without diagnosed diabetes mellitus, (iii) interventions involving dietary glycemic index, and (iv) outcomes measuring insulin resistance or insulin sensitivity.
3. **Final Inclusion**
   - We excluded non-randomized designs, observational studies, and any that involved type 1 or type 2 diabetes, gestational diabetes, or unclear methodology. Data extraction and risk-of-bias assessments were then performed on the eligible trials.
